# London Panel Committee

**Published:** 1915-04-24

**Authors:** 


### London Panel Committee.

### REDUCTION OF MEDICAL BENEFIT FUND OWING TO ENLISTMENTS.

At a meeting of the London Panel Committee on April 20 a letter was received from the London Insurance Committee asking that a proposal to pay 1s. instead of 1s. 9d. on account of capitation fees for the third quarter of 1915 be accepted by the Panel Committee. The Panel Service Sub-Committee recommended that approval be withheld.

Dr. Major Greenwood, in moving the recommendation, said that practitioners realised that the total amount of the Medical Benefit Fund must be reduced owing to the withdrawal of insured persons who had enlisted, but the proposal only to pay 1s. a quarter reduced the remuneration of individual practitioners to the old club rate of 4s. a year. To reduce the amounts received by all practitioners, whether their lists had suffered much or little by reason of enlistments, was unreasonable. An important difficulty was that some practitioners depended entirely on the sums received in respect of capitation fees, and if the Panel Committee did not concur in the reduced payment proposed by the Insurance Committee, practitioners might find their cheques withheld until the year 1915 had concluded. If this occurred, practitioners would have no legal remedy, because all payments before the end of the year were regarded as advances, and legal rights only matured when the year had ended. He felt that in honour practitioners should refuse at all costs to concur in the proposed reduction, but members of the Panel Committee should take measures to ascertain the feelings of their constituents.

The Chairman read letters from the Lambeth and Southwark Insurance Practitioners' Associations protesting against the reduction in amount of the quarterly capitation fees, urging that such a method was only necessary because the register of insured persons maintained by the Insurance Committee was not in proper order.

Dr. C. W. Hogarth moved an amendment that the Panel Committee also address a protest to the Insurance Commissioners urging them to consider the heavy burdens that would be cast upon panel practitioners as men returned from the war disabled and broken in health.

Dr. R. J. Farman, who seconded, said that the present difficulty was due to the failure of the Insurance Committee to carry out their duty of revising the register, and of the Insurance Commissioners to see that they carried out this duty.

Dr. H. H. Mills, as Chairman of the Medical Benefit Sub-Committee of the Insurance Committee, explained that a letter had been received by the Committee from the Commissioners, advising that only 64 per cent. of the sums due to practitioners should be expended. Payment having been made for the first quarter at the rate of 1s. 6d., it was felt that it would not be safe now to pay more than 1s. The occasion might very well be used to impress on the Commissioners the need for better organisation of the index registers.

The Chairman said the fact that there must be a reduction was accepted by practitioners without the slightest hesitation, but each individual should bear the share due to him.

The amendment was lost by 24 votes to 6.

The Chairman moved, and Dr. Bernstein seconded, a further amendment to substitute the following for the recommendation of the Sub-Committee :

"That the Panel Committee recognises that a reduction in the Medical Benefit Fund on account of insured persons who have enlisted is inevitable, and cheerfully accepts such reduction. The Panel Committee, however, does not concur in a general reduction being made in the sums paid to all practitioners alike, but considers that the reduction should be strictly in accordance with the reduction of each individual list."

This was carried.

Responsibility for Correcting the Register.

The Insurance Committee having forwarded to practitioners on the panel "suspense slips," with the request that the practitioner would confirm, if possible, the title to benefit of the insured persons concerned, the Panel Committee passed a resolution declaring that the onus rested upon the Insurance Committee to show that a name should be removed from a practitioner's list, and that no obligation rested upon the practitioner to submit evidence in the matter.

